# Student Pharmacists’ Perspectives Regarding a Virtually Delivered Research Proposal Course in the Doctor of Pharmacy Curriculum

**DOI:** 10.3390/pharmacy11010030

**Published:** 2023-02-05

**Authors:** David R. Axon

**Affiliations:** Department of Pharmacy Practice and Science, R. Ken Coit College of Pharmacy, The University of Arizona, 1295 N. Martin Ave., Tucson, AZ 85721, USA; draxon@arizona.edu; Tel.: +1-520-621-5961

**Keywords:** student pharmacists, student research, online teaching, educational technology, course development

## Abstract

This study aimed to assess third-year student pharmacists’ perspectives regarding a virtually delivered research proposal course. A 23-item questionnaire was distributed to third-year student pharmacists enrolled in a research proposal course over three weeks in April 2021. The questionnaire contained 15 Likert-scale items, seven descriptive items, and a free-text item for additional comments about the course. Items were summarized using descriptive statistics. Fifty-four student pharmacists (response rate = 40.9%) participated in the survey. The student pharmacists surveyed generally had a positive perception of the virtually delivered research proposal course with median scores ≥ 4 (indicating agreement) for the majority (13/15) of survey items. Students did not agree that there was no difference in their motivation to succeed in the virtual course versus an in-person course and did not agree that they were more likely to pursue a career that involves undertaking a research project. This study found that student pharmacists generally had a positive perception of a virtually delivered research proposal course. These findings offer some support for the provision of an online, virtually delivered research proposal course for student pharmacists. Further research with a larger sample of students from multiple pharmacy schools is needed to improve the generalizability of the results.

## 1. Introduction

The abrupt global emergence of the severe acute respiratory coronavirus-2 in 2019 (commonly known as COVID-19), and associated physical distancing requirements, necessitated a prompt transition to alternative methods of teaching and learning. During this pandemic, the next generation of health professionals, including pharmacists, had to learn in a virtual setting rather than the traditional in-person setting. This environment challenged pharmacy instructors to revise didactic courses, as well as Introductory Pharmacy Practice Experiences (IPPEs) and Advanced Pharmacy Practice Experiences (APPEs), for virtual learning [1]. The Accreditation Council for Pharmacy Education (ACPE) Accreditation Standards broadly requires students to be exposed to research skills such as the evaluation of the scientific literature, implementing solutions, and advancing creative thinking to reach professional goals, yet the methods employed to give student pharmacists a research-focused education vary across accredited institutions [2]. Research courses grant student pharmacists the skills to apply new evidence to pharmacy practice and boost their confidence in research [3]. Doctor of Pharmacy (PharmD) programs are encouraged to cultivate self-directed students that can assimilate and apply vast amounts of research. These skills are important because pharmacists are responsible for synthesizing reliable and valid healthcare research and providing it to the patients and communities they serve. This is all the more important during a pandemic, when there is rapidly evolving and sometimes contrasting information available [4,5,6].

Student pharmacists enrolled at The University of Arizona R. Ken Coit College of Pharmacy accomplish these ACPE domains by designing and then conducting their own pharmacy research project through a series of required research courses. The first course in this series (the research proposal course) is the focus of this paper. In this required two-credit course offered in the Spring semester of the third year, student pharmacists form teams or work independently, choose a project advisor, and are taught how to develop a research proposal for a project to they would like to pursue. After this proposal is approved by all necessary parties (course coordinator, project advisor, institutional review board, etc.), they may begin their project. Students then take two required credits of independent study in both the Fall and Spring semesters of their fourth year (a total of four credits of independent study) to complete their project, make and present a poster of their findings, and produce a research report. The University of Arizona R. Ken Coit College of Pharmacy has a primary campus in Tucson, Arizona, and a satellite campus in Phoenix, Arizona. This dual campus model of pharmacy education has become commonplace alongside the advancement of live video conferencing software [7,8]. In general, an instructor at the main campus (Tucson) teaches in person while sending a live video stream to the satellite campus (Phoenix). Occasionally, the roles are reversed when an instructor at the satellite campus sends a live video stream to the main campus.

In Spring 2021, physical distancing and stay-at-home mandates imposed due to COVID-19 forced this research proposal course to transition from its usual in-person format to an online, virtual format using Zoom technology (Zoom Video Communications, Inc, San Jose, CA, USA). The same course assignments were used in the online course as were used in the previous in-person course. The instructor’s goal was to maintain the same level of student engagement with the content using a virtual format. Previous scientific literature has described best practices for implementing remote learning with professional students and described how students interact with online and electronic learning tools [9,10,11]. Previous research has also found that campus type (e.g., main campus or satellite campus) does not influence academic performance in skill-based courses taught across multiple sites [12].

The recent literature describes the effectiveness of online learning generally [9,10] and in specific courses such as pharmacokinetics and communications [13,14]. However, there is limited information describing student pharmacists’ perceptions regarding a virtually delivered research proposal course, such as the one described in this paper. Given that the nature of conducting a research project is somewhat different from didactic learning, it is important to understand if students’ perceptions are similar or different from what is already known about online learning. Therefore, the objective of this study was to assess third-year student pharmacists’ perspectives regarding a virtually delivered research proposal course.

## 2. Methods

Students were eligible to participate in this cross-sectional survey if they were third-year student pharmacists enrolled in the University of Arizona R. Ken Coit College of Pharmacy research proposal course in Spring 2021 (*N* = 132). The goal of this two-credit course was to prepare a research proposal that can be completed during the final year of the pharmacy program. All students enrolled in this course had previously completed six credits of coursework in drug literature evaluation courses (e.g., statistics, study design), and three credits of coursework in quality improvement that included a year-long team-based quality improvement project. The research proposal course class met virtually via Zoom^®^ for two hours each week throughout the entirety of the Spring 2021 semester. In the class, students decided if they wanted to work independently or form a team of up to five students. Students identified their project advisors, who are often practicing pharmacists and/or university faculty. Students were required to meet frequently with their project advisor to seek their input and feedback on the project proposal. All course materials were provided to students via the online course management software at the start of the course. These materials included a template proposal and example proposals to guide the development of their proposal. Each week in class, the instructor focused on one aspect of the proposal and guided students through a sequence of tasks to develop their proposal. In class, after an explanation and example of the week’s tasks, the remainder of the class time was allocated for students to work on their proposals and seek feedback from the instructor as necessary. Students were then required to complete those tasks by the end of the week. Students were able and encouraged to work ahead of the course schedule to give themselves more time to conduct their studies or complete their studies early. Toward the end of the semester, the instructor reviewed and provided feedback on a draft of the proposal for students to act on, such that students had a complete proposal for an appropriate project by the end of the semester. After the proposal was approved by the course coordinator and project advisor, students obtained Institutional Review Board (IRB) approval as necessary and began their project. The content and assignments used in this online version of the course were the same as those used when the course was taught in person. However, there were necessary practical differences such as all materials being provided electronically and the instructor not being able to move around the classroom to help students independently. Students were able to use their own Zoom rooms to work independently on their projects both during and outside class time. An outline of the course content and time spent in each area is provided in Table 1.

A 23-item questionnaire (Appendix A (Table A1)) was designed specifically for this study using REDCap (Research Electronic Data Capture) hosted at The University of Arizona. REDCap is a secure, web-based software platform designed to support data capture for research studies, providing (1) an intuitive interface for validated data capture; (2) audit trails for tracking data manipulation and export procedures; (3) automated export procedures for seamless data downloads to common statistical packages; and (4) procedures for data integration and interoperability with external sources [15,16]. The first section of the questionnaire asked students to rate their level of agreement with 15 statements using a six-point Likert Scale (1 = strongly disagree, 2 = disagree, 3 = somewhat disagree, 4 = somewhat agree, 5 = agree, 6 = strongly agree). The first four Likert Scale items evaluated students’ perceived ability and motivation to succeed in the course and communicate with the instructor. The next six items assessed student perceptions of whether they gained the skills from the ACPE learning domains that the course was designed to meet. The next three Likert Scale items assessed student perceptions of the requisite research projects in their curriculum, a career in research, and how well the course prepared them for such a career. The final two Likert Scale items assessed student perceptions of the class structure. The next section of the questionnaire consisted of seven demographic and descriptive items about student characteristics, experience, and goals. The questionnaire ended with a free-text item for participants to provide any additional comments they had about the course. An initial draft of the questionnaire was developed based on items from Darr et al. [17], with further revisions made until the instrument was deemed to have appropriate face validity by the researcher.

Data were collected over a three-week period in April 2021. This timeframe was chosen so that students had experienced most of the course and would be able to provide feedback on it, without encroaching on the examination period at the end of the semester. An email containing information about the study and a link to participate in the online questionnaire was sent to eligible participants. All students were informed that their participation was voluntary and that their responses would be recorded anonymously. A reminder email was sent after one week, and a further reminder email was sent after two weeks. Data collection stopped at the end of the third week. Data were exported from REDCap and analyzed with SAS (V9.4., Cary, NC, USA). The Likert Scale items were summarized using medians with interquartile ranges, nominal items were summarized using frequencies with percentages, and items relating to participants’ self-identified experience with videoconferencing and preparedness for the course were summarized using means with standard deviations. This study was reviewed and approved by the University of Arizona Institutional Review Board.

## 3. Results

In sum, 54 of the 132 (40.9%) enrolled students submitted completed questionnaires. The demographic and descriptive characteristics of study participants are shown in Table 2. Most participants were female (61.1%), had not previously conducted research (72.2%), attended the main (Tucson) campus (59.3%), did not anticipate holding a position that required conducting research upon graduation (81.5%), and were working as part of a group for their project (94.4%).

Participants’ level of agreement with survey items are shown in Table 3. The median level of agreement was 4 or above (i.e., indicating at least some level of agreement) for most items, except for “there was no difference in my motivation to succeed in this virtual course versus an in-person course” (median = 3.5) and “after taking this course, I am more likely to pursue a career that involves undertaking a research project” (median = 3.0).

A total of 18 responses were received from the open-text item, of which 9 contained substantive content (i.e., were not responses of “thank you”, “no comment”, “not applicable”, etc.). Three responses requested more time for choosing a project or for submitting assignments, another three responses referred to students enjoying the virtual format or that the virtual format worked well for this course, two comments alluded to students’ preferences for in-person courses, and one comment mentioned that the student enjoyed the course, and that the instructor was helpful.

## 4. Discussion

The main finding from this study was that the student pharmacists surveyed generally had a positive perception of the virtually delivered research proposal course, as indicated by median scores of 4 or above for the majority (13/15) of survey items. This finding indicates the perceived importance of research experiences for student pharmacists and suggests the non-inferiority of a virtually delivered research proposal course. The findings from this study are discussed below in more detail.

This study found that student pharmacists agreed that there was no difference in their ability to succeed in the course or communicate with the instructor in the virtual setting. This aligns with other studies that found that student performance was unaffected in virtual laboratory courses [12], elective immunization course [18], and pharmacology courses [19], and suggests that virtual delivery may be appropriate for a research proposal course as well. Interestingly, students did not agree that there was no difference in their motivation to succeed in the virtual course compared to an in-person course. However, this study did not assess whether there was more motivation or less motivation to succeed in the virtual course. Students may be more motivated because they have greater flexibility to complete their tasks, or perhaps they may be less motivated to complete their assignments without an in-person instructor. All students had a dedicated research project advisor and experienced course coordinator who met with each group periodically throughout the semester, and students were advised that they were able to contact the course coordinator to discuss their research proposal as necessary. However, further effort is needed to investigate and implement factors that could be used to help motivate students to succeed in this virtually delivered course. Although students perceived that there was no difference in their ability to succeed in a virtually delivered research proposal course, the finding that they perceived that there was a difference in their motivation to succeed in a virtually delivered research proposal course poses questions about whether lower motivation is a hindrance to virtually delivered instruction. Previous work has found that students learning in self-paced systems such as virtual courses receive diminished external regulatory cues from their peers and instructor, which is associated with lower motivation, leading to procrastination [20]. Lower engagement in the course could ultimately lead to more time being needed to complete assignments and/or a decline in the quality of the project. Historically, students have identified lower satisfaction with virtual settings, reporting drawbacks such as nonsynchronous interaction and lower quality instructor engagement, though it has been observed that students also conclude that the experience is ultimately similar to in-person courses [12,18,21]. More study in this area is warranted, as there is limited literature regarding how long-term virtually delivered courses affects student pharmacists’ (or other students’) perspectives of the environment and their ability and motivation to succeed in the course.

This study also found that student pharmacists expressed a strong level of agreement that research was an important part of their pharmacy education and agreed that they were better prepared to conduct research after taking this course. However, this study also found that student pharmacists did not agree that they were more likely to pursue a career that involved conducting research projects. This phenomenon has previously been reported among student pharmacists in research courses [3]. Research benefits student pharmacists by improving skills essential for evidence-based pharmacy practice and offering competitive experience for desired positions post-graduation [22,23,24]. However, the majority do not foresee themselves actively conducting research, perhaps suggesting they intend to utilize the scientific literature but do not intend to actively contribute to it. More research, perhaps using focus groups, is needed to clarify and establish student pharmacists’ perspectives on how their research education correlates with their future research needs and plans. With the role of the pharmacist growing increasingly complex, the need for pharmacists to be both creators and critical consumers of research is expanding [25,26]. As the number of student pharmacists advancing to residency and research increases, it is reasonable for institutions to offer formal research experiences to student pharmacists. The existing literature suggests that research experiences offered to student pharmacists through required or elective courses and projects can benefit both the mentor (usually a faculty member or preceptor) and students [3,27,28].

Furthermore, this study found that students reported that they liked the structure of the course, which enabled students to work on their proposal at their own pace and use class time to engage with the instructor, rather than a lecture-style course and needing to ask questions outside of class. This resembles the flipped classroom mode of instruction, whereby students had access to course materials to review ahead of the class and then utilized most of the class time to employ active learning techniques and work on their research projects at their own pace. This approach also enabled students to work ahead of schedule as desired. In a flipped classroom, students prepare for class by reading and/or watching pre-recorded content, and class time is then devoted to applying new knowledge through interactive activities such as problem-solving and discussion [29]. Flipped classroom designs are being commonly adopted in health care education, and previous work has suggested that their adoption may be associated with a minor gain in student pharmacist knowledge when compared to lecture-based courses [30].

The findings from the free-text item were limited and provided little additional insight specifically about the virtual nature of the course. Further work using qualitative interviews or focus groups would be welcome to explore this area further.

This study had some limitations. The necessary assumption inherent to survey-based research is that participants understood and responded accurately to survey items. Although the instrument was developed based on the existing literature and deemed to have appropriate face validity, further validation assessments were not undertaken. Future studies should involve a full validation assessment of the instrument to improve the credibility of the findings. This study had a small sample size of 54 students from one college of pharmacy, and therefore the findings may not be generalizable to all student pharmacists. The response rate of approximately 40% also limits the representativeness of the findings. This study had no comparison or control group and students had not received this course in any other modality (e.g., in-person), and thus these findings are based on students’ perceptions (subjective) and not any objective data. Future studies using large sample sizes of student pharmacists from multiple schools of pharmacy are needed to improve the external validity of the findings. Future studies could also be undertaken after the COVID-19 pandemic to assess if students’ perceptions of a virtually delivered research proposal course remain the same now that in-person learning is again available.

## 5. Conclusions

This cross-sectional observational study found that third-year student pharmacists at the University of Arizona generally had a positive perception of a virtually delivered research proposal course. Students generally liked the design of the course and agreed that it offered them opportunities to develop various skill sets. Student pharmacists perceived that they were able to succeed in the course and communicate with the instructor in the virtual setting. Students also perceived that research projects were an important part of their pharmacy education and were prepared to conduct a research project after taking the course. However, students typically did not agree that there was no difference in their motivation to succeed in the virtual course versus an in-person course and did not agree they were more likely to conduct research in the future. These findings provide some evidence to support conducting research projects in the pharmacy curricula and that students perceived it as appropriate to offer a research proposal writing course virtually. However, further research with a larger sample size and student cohorts from other schools of pharmacy is needed to produce more generalizable findings.

## Figures and Tables

**Table 1:** Overview of course content and time spent on each area.

Course Content: Topic	Time Spent
Course overview, finding a project and project advisor, and reviewing research proposal templates	2 h
Writing the problem statement, literature review, purpose statement, and specific aims/hypotheses	2 h
Literature searching	2 h
Writing the methods section	4 h
Developing data collection forms and a data dictionary	4 h
Timeline, budget, and references	2 h
Human Subjects/IRB	4 h
Reviewing proposal drafts	4 h
Preparing for fourth year research project and APPE rotations	6 h

Abbreviations: IRB = institutional review board; APPE = advances pharmacy practice education.

**Table 2:** Third-year student pharmacist demographic and descriptive characteristics (*N* = 54).

Variable	Result
Female gender, *N* (%)	33 (61.1)
Had no previous research experience before writing the research proposal, *N* (%)	39 (72.2)
Attend main (Tucson) campus, *N* (%)	32 (59.3)
Do not anticipate a position conducting research after graduation, *N* (%)	44 (81.5)
Working on research project as part of a team, *N* (%)	51 (94.4)
Self-identified videoconferencing experience ^1^, mean (standard deviation)	7.8 (1.7)
Self-identified level of preparation for course ^2^, mean (standard deviation)	6.4 (2.1)

^1^ Evaluated with a ten-point scale where 0 = not all experienced and 10 = extremely experienced. ^2^ Evaluated with a ten-point scale where 0 = not all prepared and 10 = extremely prepared.

**Table 3:** Third-year student pharmacist’s level of agreement with survey items (*N* = 54).

Survey Statement	Median (IQR)
There was no difference in my ability to succeed in this virtual course versus an in-person course.	4.5 (3.0)
There was no difference in my motivation to succeed in this virtual course versus an in-person course.	3.5 (3.8)
There was no difference in my ability to communicate with the instructor in this virtual course versus an in-person course.	4.5 (3.0)
There was no difference in my motivation to communicate with the instructor in this virtual course versus an in-person course.	4.0 (3.0)
The virtual workshop design of the course is an innovative teaching strategy.	4.5 (1.8)
The virtual workshop design of the course is appropriate for the Doctor of Pharmacy curriculum.	5.0 (1.0)
The virtual workshop design of the course aided in the development of my self-directed learning skills.	5.0 (1.0)
The virtual workshop design of the course aided in the development of my problem-solving skills.	4.0 (1.0)
The virtual workshop design of the course aided in the development of my time management skills.	4.0 (2.0)
The virtual workshop design of this course aided in the development of my videoconferencing skills.	4.0 (1.0)
Completing a research project is an important part of my Doctor of Pharmacy education.	5.0 (2.0)
After taking this course, I am more likely to pursue a career that involves undertaking a research project.	3.0 (2.0)
After taking this course, I am better prepared to pursue a career that involves undertaking a research project.	4.0 (2.0)
I prefer having the proposal writing instructions and examples at the start of the semester so I can work on the proposal at my own pace rather than having instructions and examples provided as lectures each week in class.	5.5 (2.0)
I prefer using class time to discuss any questions I have about my project with the instructor rather than asking questions outside of class or via email.	5.0 (2.8)

All statements were evaluated on six-point Likert scale where 1 = strongly disagree, 2 = disagree, 3 = somewhat disagree, 4 = somewhat agree, 5 = agree, 6 = strongly agree. Abbreviations: IQR = interquartile range.

## Data Availability

Data are available from the author on request.

## Appendix A

**Table A1:** Questionnaire used to collect data from third-year student pharmacists’ regarding a virtually delivered research proposal course.

Please Rate Your Level of Agreement with the Following Statements:						
	Strongly Disagree	Disagree	Somewhat Disagree	Somewhat Agree	Agree	Strongly Agree
There was no difference in my ability to succeed in this virtual course versus an in-person course.						
There was no difference in my motivation to succeed in this virtual course versus an in-person course.						
There was no difference in my ability to communicate with the instructor in this virtual course versus an in-person course.						
There was no difference in my motivation to communicate with the instructor in this virtual course versus an in-person course.						
The virtual workshop design of the course is an innovative teaching strategy.						
The virtual workshop design of the course is appropriate for the Doctor of Pharmacy curriculum.						
The virtual workshop design of the course aided in the development of my self-directed learning skills.						
The virtual workshop design of the course aided in the development of my problem-solving skills.						
The virtual workshop design of the course aided in the development of my time management skills.						
The virtual workshop design of this course aided in the development of my videoconferencing skills.						
Completing a research project is an important part of my Doctor of Pharmacy education.						
After taking this course, I am more likely to pursue a career that involves undertaking a research project.						
After taking this course, I am better prepared to pursue a career that involves undertaking a research project.						
I prefer having the proposal writing instructions and examples at the start of the semester so I can work on the proposal at my own pace rather than having instructions and examples provided as lectures each week in class.						
I prefer using class time to discuss any questions I have about my project with the instructor rather than asking questions outside of class or via email.						
Please provide any comments you have about this virtual research course:						
Please enter the following demographic and background information						
Gender					Male	
					Female	
Have you worked on any other research project (other than the Quality Improvement project) before your Doctor of Pharmacy research project?					Yes	
					No	
Which University of Arizona campus do you attend?					Tucson	
					Phoenix	
Are you working independently or as part of a group for your Doctor of Pharmacy research project?					Independent	
					Group	
Following graduation, do you anticipate holding a position that will involve conducting research?					Yes	
					No	
On a scale of 0 (not at all experienced) to 10 (extremely experienced), how experienced are you with using videoconferencing technologies for your coursework?					0–10	
On a scale of 0 (not at all) to 10 (extremely prepared), how well did previous research-focused courses (e.g., drug literature evaluation, statistics, study design, quality improvement, etc.) prepare you for writing your senior research project?					0–10	
